# Coping strategies of HIV-affected households in Ghana

**DOI:** 10.1186/s12889-015-1418-x

**Published:** 2015-02-21

**Authors:** Amos Laar, Abubakar Manu, Matilda Laar, Angela El-Adas, Richard Amenyah, Kyeremeh Atuahene, Dave Quarshie, Andrew Anthony Adjei, Isabella Quakyi

**Affiliations:** Department of Population, Family, & Reproductive Health, School of Public Health, College of Health Sciences, University of Ghana, P. O. Box LG 13, Legon Accra, Ghana; School of Dietetics and Human Nutrition, McGill University, Montreal, Canada; Ghana AIDS Commission, Accra, Ghana; School of Management, Blekinge Tekniska Högskola, SE-371 79 Karlskrona, Sweden; Department of Pathology, University of Ghana Medical School, College of Health Sciences, University of Ghana, Accra, Ghana; Department of Biological, Environmental, and Occupational Sciences, School of Public Health, University of Ghana, Accra, Ghana

**Keywords:** Coping strategies, HIV, HIV-affected households, Food insecurity, Ghana

## Abstract

**Background:**

HIV and negative coping mechanisms have a cyclical relationship. HIV infections may lead to the adoption of coping strategies, which may have undesired, negative consequences. We present data on the various coping mechanisms that HIV-affected households in Ghana resort to.

**Methods:**

We collected data on coping strategies, livelihood activities, food consumption, and asset wealth from a nationally representative sample of 1,745 Ghanaian HIV-affected households. We computed coping strategies index (CSI), effective dependency rate, and asset wealth using previously validated methodologies.

**Results:**

Various dehumanizing coping strategies instituted by the HIV-affected households included skipping an entire day’s meal (13%), reducing portion sizes (61.3%), harvesting immature crops (7.6%), and begging (5.6%). Two-thirds of the households were asset poor. Asset-poor households had higher CSI than asset-rich households (*p* <0.001). CSI were also higher among female-headed households and lower where the education level of the household head is higher. Households caring for chronically ill members recorded higher CSI in comparison with their counterparts without the chronically ill (*p* < 0.05).

**Conclusions:**

Institution of degrading measures by HIV-affected households in reaction to threat of food insecurity was prevalent. The three most important coping strategies used by households were limiting portion size (61.3%), reducing number of meals per day (59.5%) and relying on less expensive foods (56.2%). The least employed strategies included household member going begging (5.6%), eating elsewhere (8.7%) and harvesting immature crop (7.6%).

Given that household assets, and caring for the chronically ill were associated with high CSI, a policy focusing on helping HIV-affected households gradually build up their asset base, or targeting households caring for chronically ill member(s) with conditional household-level support may be reasonable.

## Background

Human immunodeficiency virus (HIV) without a doubt is a grave public health and development problem in sub-Saharan Africa. The Joint United Nations Programme on HIV/AIDS (UNAIDS) in 2012 estimated that more than two-thirds of the over 35 million people living with HIV worldwide live in sub-Saharan Africa [1]. This region hosts only 12% of the world population [2]. The fight against HIV/AIDS is being pursued through interventions to stop the spread of the virus, and prolong the lives of those infected through the use of antiretroviral therapy (ART). Significant successes have been made in this drive. The most recent estimates suggest that the total number of new HIV infections in sub-Saharan Africa has dropped significantly [1,2]. The HIV epidemic has been stable in Ghana over the past half-decade. Indeed, linear trend analysis of prevalence data from 2000 to 2013 shows that HIV situation in Ghana has declined [3]. The most recent prevalence data shows an estimated national adult HIV prevalence of 1.3% [4].

There has been an increased access to ART globally [1] and locally [5]. Access to treatment commodities have been shown to lead to a reduction in the number of AIDS-related deaths [2,6,7]. In spite of these efforts, one major challenge many HIV-affected individuals and households in sub-Saharan Africa grapple with is food insecurity. Studies have shown that HIV exacerbates the vulnerability of affected families to food insecurity, leading to hunger and malnutrition [8,9]. For instance, a longitudinal study in Uganda among HIV-infected individuals had shown that severe food insecurity was associated with worsened quality of life [10]. Indeed, scholars have previously provided elucidation on the relationship between HIV and food insecurity. The relationship is complex and intertwined in a vicious cycle, with each worsening vulnerability and thus exacerbating the severity of the other [10,11]. Food insecurity heightens susceptibility to HIV exposure and infection; HIV on the other hand increases vulnerability to food insecurity. This relationship is often compounded by low income, resulting in profound consequences on health and nutritional status. Households that suffer from food insecurity due to poverty are malnourished prior to infection [11]. As a disease, HIV’s impact on malnutrition as a result of its effect on the infected individual’s metabolism, ingestion and digestion of food has long been clarified [12-15].

HIV also disrupts livelihoods as infected persons often lose the ability to work and generate income [16]. In addition, the propensity for uninfected family members to contribute economically to the household income basket is seriously affected due to the burden of care for the infected person(s). For instance, it is reported that caring for an individual with AIDS in sub-Saharan Africa can deplete as much as one-third of a family’s monthly income [17]. This situation feeds into the vicious cycle of HIV and food insecurity described above.

Sometimes described as a syndemic^a^, the relationship between HIV and food insecurity often causes individuals and households to adopt coping strategies to maintain the status quo. Studies have demonstrated that such strategies are often negative, undesired, unsustainable and often irreversible [18,19]. Strategies that have often been adopted include sale of assets, taking children out of school, migrating and engaging in transactional sex [11,16,20]. Some authorities posit that these coping strategies may bring short-term relief, but increases the risk of exposure to HIV. Destitution and despair brought on by negative coping behavior may increase the risk that a person will resort to trading unprotected sex for food [21,22].

This background shows that there is a growing body of literature on HIV, food insecurity and negative coping mechanisms in sub-Saharan Africa, with majority of the studies originating from Southern African countries [10,16,20,23-25]. Little is known about other sub-Saharan African countries, including Ghana. Most HIV-related studies in Ghana have largely focused on epidemiology, behavioral, social and psychological aspects of the disease. There is paucity of data on how HIV-infected individuals and affected households address their economic needs amid living with the disease. This paper presents data on the various coping strategies of a nationally representative sample of 1,745 Ghanaian HIV-affected households.

## Methods

### Design and methodology

The study was cross-sectional in design involving a nationally representative sample of 1,745 HIV-affected households. The sampling procedure of this national representative cross-sectional survey is detailed elsewhere [26]. Respondents were sampled from all of the ten regions in Ghana. Regional sample sizes were adjusted according to the size of each region in terms of number of PLHIV, and the proportion of PLHIV on ART. This paper is based on a total of 1,745 questionnaires representing 1,745 HIV-affected households, which were retained after data cleaning. After selecting households through a systematic random sampling procedure, PLHIVs from selected households were approached to schedule household interviews using the assessment questionnaire. Most of the interviews took place in the interviewee’s home.

#### Assessment of coping strategies

To explore the concept of coping, a simple numeric score, referred to as coping strategy index (CSI) was constructed using a series of questions about how households cope with a shortfall in food for consumption. The variables considered address the recurrent situation faced by the household, and the coping strategies adopted to deal with food insecurity. The variables include limiting portion size at mealtimes, reducing number of meals eaten per day, skipping an entire day’s meal, borrowing food or relying on help from friends or relatives, relying on less expensive or less preferred foods, hunting/gathering unusual types or amounts of wild food, harvesting immature crops (e.g. green maize) and sending household members to eat elsewhere. Given a recall period of three months, respondents were asked to indicate how frequently their households resorted to using one or more of the above-mentioned strategies in order to have access to food. Study subjects’ responses to questions on these variables allow an assessment of the frequency as well as the severity of actions. The questions on coping strategies fall into two types. Firstly, they address the recurrent situation faced by the household, and the coping strategies adopted to deal with food insecurity. Also considered are changes in household strategies in response to recent difficulties, for example by asking whether the household has recently reduced the number of meals consumed per day, or purchased lower cost ingredients. It is worthy of note that the questions we used in constructing the CSI were adopted from a standard and previously validated tool [26]. An illustration of how responses are transformed into the CSI is shown in Table 1.

**Table 1:** **Computation of the coping strategy index: an illustration**

**Coping strategies**								
		**Never**	**Seldom (1-3 days/month)**	**Sometimes (1-2 days/week)**	**Often (3-6 days a week)**	**Daily**		
	**Relative frequency score (A)**	**0**	**0.5**	**1.5**	**4.5**	**7.0**	**Severity weight (B)**	**Score (AxB)**
E1	Skip entire days without eating?	1	2	3	4	5	8.0	0
E2	Limit portion size at mealtimes?	1	2	3	4	5	2.0	9
E3	Reduce number of meals eaten per day?	1	2	3	4	5	2.0	1
E4	Borrow food or rely on help from friends or relatives?	1	2	3	4	5	4.0	0
E5	Rely on less expensive or less preferred foods?	1	2	3	4	5	2.0	1
E6	Purchase/borrow food on credit?	1	2	3	4	5	4.0	6
E7	Gather unusual types or amounts of wild food/hunt?	1	2	3	4	5	8.0	0
E8	Harvest immature crops (e.g. green maize)?	1	2	3	4	5	8.0	0
E9	Send household members to eat elsewhere?	1	2	3	4	5	4.0	0
E10	Send household members to beg?	1	2	3	4	5	8.0	0
E11	Reduce adult consumption so children can eat?	1	2	3	4	5	6.	0
E12	Rely on casual labour for food?	1	2	3	4	5	2.0	0
	TOTAL HOUSEHOLD SCORE	$={\displaystyle \sum_{k=0}^n\mathrm{A}\times \mathrm{B}=17.0}$						

*Table legend: Table shows the relative frequency score, the severity weight, the individual score and the total household score of a randomly selected household. The total household score (∑A×B); or the summation of the products of each raw score/relative frequency score and the severity weight for each strategy) is defined as the CSI for that particular household and is 17.0.*

#### Computation of household asset wealth

Asset wealth was assessed in the survey through questions on the type of asset owned by the household – these assets fall into two general categories, one describing the standard of living of the household (assets such as chairs, tables), and the other associated with income earning possibilities (items such as pop-corn machines, telephone booths or hairdryers etc). Households were split into three broad classes, according to how many different types of asset they own – “asset poor”; “asset medium”; and “asset rich”. Details on the construction of the asset wealth variable are given in a previously published UNAIDS technical report [27].

#### Computation of effective dependency rate

The effective dependency rate measures the share of total household members that is below or above working age plus those of working age who are chronically ill. The study defined the chronically ill as a person who by reasons of his/her HIV status or any other health condition or disability experiences a diminished level of functioning relative to primary level of daily living. To qualify, such a person must have been in that state for a minimum of six months. For every household, the numbers of these three categories of members were subtracted from the total household size and expressed as a percentage. Details of the computations are available in [27].

### Data management and analysis

All data were entered in Epi Data (Version 3.1) and later exported to IBM SPSS Statistics for Windows (Version 20.0) for analysis. We used univariate analysis to generate descriptive tabulations for key variables. Statistics presented with accompanying 95% confidence intervals are derived from such descriptive analysis. Bivariate analysis produced unadjusted associations between CSI and some selected household demographic or health attributes. Two-sided test of statistical significance was performed and P value <0.05 was used to denote statistical significance. All analyses were performed using IBM SPSS Statistics for Windows, Version 20.0.

### Ethical considerations

This study protocol was reviewed and approved by the Ghana Health Service Ethical Review Committee to ensure that the study adhered to both local and international standards for protecting the rights and safety of human subjects in research. Informed consent was obtained from all participants after the objectives and the methodology of the study was explained to them. In addition, participants were assured of privacy and confidentiality.

## Results

We introduce our findings with a presentation of the demographic profiles, the sources of livelihood, household asset wealth, and effective dependency rate of the households.

### Demographic profiles and household composition

The majority of the respondents were females (75%). The proportion of female-headed HIV-infected households is almost equal to male-headed households although there are notable regional variations. The average size of households differed by household headship. Those headed by men had on average three members, compared to two for female-headed households. Forty-one percent of respondents are married, 15% are divorced, and 20% are widowed. Nearly 72% have attained at least a primary school education. Nationally, 16.3% of the main respondents were chronically energy deficient (defined as BMI < 18.5 kg/m2). The rate was highest in the Central Region (27.5%) and lowest in the Upper East Region has. Details of the regional distribution of this statistic are given in Table 2. We have also presented in Table 2 some of the above attributes by sex of household health.

**Table 2:** **Socio-demographics, and other attributes of household members (N = 1745)**

**Attribute**	**Proportion N = 1745**	**Sex of household head**			
		**Male**		**Female**	
		**N**	**(%)**	**N**	**(%)**
**Sex of main head of household**					
Female	50.6				
Male	49.4				
**Sex of main respondent**					
Female	74.7	457	53.0	847	95.9
Male	25.3	405	47.0	36	4.1
**Age of main respondent**					
15 years or younger	2.2	17	2.0	21	2.4
16 to 19 years	1.5	8	.9	18	2.0
20 to 39 years	49.4	402	46.6	460	52.1
40 to 59 years	42.1	383	44.4	351	39.8
60 years or older	4.9	52	6.0	33	3.7
**Marital status of main respondent**					
Divorced	15.2	80	9.3	186	21.1
Married	40.6	571	66.2	138	15.6
Separated	7.1	28	3.2	96	10.9
Never married	16.6	100	11.6	190	21.5
Widowed	20.4	83	9.6	186	21.1
**Educational level of main respondent**					
Never been to school	27.7	212	24.6	271	30.7
Primary	30.3	251	29.1	277	31.4
Secondary	35.6	327	37.9	294	33.3
Tertiary	5.9	69	8.0	34	3.9
No information on education	0.6	3	0.3	7	0.8
**Main source of livelihood of households**					
Salaried worker	11.0	139	16.1	52	5.9
Cash crop production	17.0	177	20.5	119	13.5
Casual labor	5.0	41	4.8	46	5.2
Begging	0.5	5	.6	8	.9
Livestock	0.5	7	.8	3	.3
Skilled trade	12.0	117	13.6	84	9.5
Petty trade	23.0	116	13.5	283	32.0
Remittance	10.0	66	7.7	112	12.7
Food assistance	0.5	6	.7	11	1.2
**Dependency rate**					
50% or less	73.8	330	60.6	315	56.5
Greater than 50%	26.2	215	39.4	243	43.5
**Asset wealth**					
Poor	1022	462	53.6	560	63.4
Medium	696	386	44.8	310	35.1
Rich	27	14	1.6	13	1.5
**Regional distribution of asset poor households**					
National	67.2				
Ashanti region	60.2				
Brong Ahafo region	66.7				
Central region	37.2				
Eastern region	84.2				
Greater Accra region	53.3				
Northern region	80.9				
Upper East region	72.5				
Upper West region	54.1				
Volta region	90.4				
Western region	71.6				
**Chronic energy deficiency (BMI < 18.5 kg/m2) by region**					
National	16.3				
Ashanti region	18.4				
Brong Ahafo region	7.6				
Central region	27.5				
Eastern region	20.5				
Greater Accra region	21.4				
Northern region	20.4				
Upper East region	8.3				
Upper West region	7.4				
Volta region	16.9				
Western region	10.9				

### Sources of livelihood, household asset wealth, and effective dependency rate

The main sources of household income are petty trading, cash crop production, skilled trade and casual labor. Other sources include remittances and income from wage employment. Asset wealth is assessed in the survey through questions on the type of asset owned by the household–these assets fall into two general categories, one describing the standard of living of the household (assets such as chairs, tables), and the other associated with income earning possibilities (items such as pop-corn machines, telephone booths or hairdryers). About two-thirds of HIV affected households were asset poor. With the exception of Central region with an asset poor rate of 37%, all other regions recorded higher proportions of asset poor households; these ranged from 72% - 90% (Table 2). The national effective dependency rate of 48.5% does not compare with dependency rates of some of the regions (Table 2). Northern region has the highest proportion of effective dependents per household (75%) - close to 30 percentage points higher than the national average. The distant second is the Eastern region (65%). The Central, Upper east, Upper west, and Western regions each recorded a rate close to the national average. The national capital (Greater Accra region) recorded the lowest number of effective dependents per household – 40% (regional data not shown).

### Coping strategies instituted by HIV-affected households

**Figure 1:**
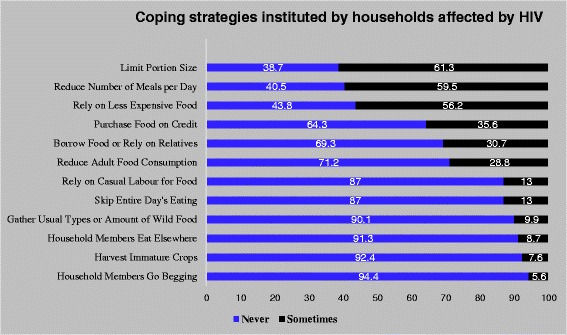
**Coping strategies instituted by household affected by HIV.** Legend: Figure 1 explores the concept of coping. Prevalence of various short-term different behavioral responses (food consumption coping strategies) in response to threat of, or actual food insecurity are presented. The strategies range from limiting portion size at mealtimes (61.3%) to sending household members to go begging (5.6%).

Our data show that households affected by food insecurity employ short-term different behavioral responses (food consumption coping strategies) to manage food shortages in the household. The frequency with which households adopted various coping strategies ranged from 5.6% to 61.3% (Figure 1). The three most important coping strategies used by households to cushion food insufficiency were limiting portion size (61.3%), reducing number of meals per day (59.5%) and relying on less expensive foods (56.2%). Conversely, the least employed strategies included household member going begging (5.6%), eating elsewhere (8.7%) and harvesting immature crop (7.6%). It is worth noting that most households do not use a single strategy, but a combination of strategies.

### Coping strategy index and its relationship with some selected attributes of HIV-affected households

**Figure 2:**
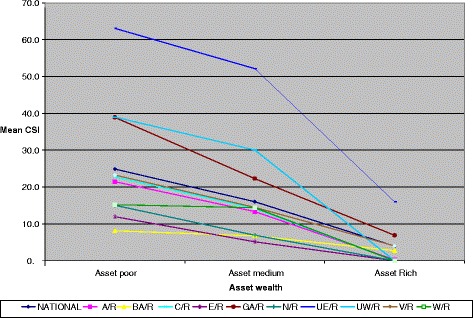
**Household coping strategies index by asset wealth and region.** A/R = Ashanti Region; BA/R = Brong Ahafo Region; C/R = Central Region; E/R = Eastern Region; N/R – Northern Region; UE/R = Upper East Region; UW/R = Upper West Region. An analysis of variance testing the differences in CSI by asset wealth revealed that both Asset Rich and Asset Medium household recorded significantly lower CSI (p < 0.001) in each case.

We explored the concept of using the coping strategy index (CSI). The CSI is a proxy measure of relative food insecurity in a household; with lower scores reflecting better household food security situation. The national average CSI was 21, and this was three times lower than that of the Upper East region (Figure 2). The CSI for HIV affected households is lowest in Brong Ahafo region (Figure 2). The CSI was further applied to selected socio-demographic explanatory variables such as household asset wealth, headship of household, educational level of household head. At both the national and regional levels, logical relationships were observed. We observed an inverse relationship between asset ownership and CSI. Asset rich households have the lowest CSI, and the opposite is true for asset poor households (Figure 2). There were clear patterns between CSI scores and certain household characteristics. One such characteristic is headship of household (female versus male). The CSI scores were significantly higher among female-headed households (p < 0.05). The level of education of household heads was also associated with the CSI. The CSI was generally lower where the education level of the household head is higher (Table 3).

**Table 3:** **Distribution of household coping strategy index by some selected attributes**

**CSI**	**Mean**	**95% confidence interval for the mean**		
		**Lower limit**	**Upper limit**	**Remarks**
**Sex of household head**				
Male	18.2	16.0	20.4	SSD
Female	24.4	22.0	26.8	
**Educational level of household head**				
Never been to school	27.7	24.1	31.2	NSSD
Primary level education	20.0	17.2	22.8	
Secondary level education	18.4	15.9	21.0	
Tertiary level education	17.0	10.7	23.3	
**Presence of orphan(s) in the household**				
No orphan	21.2	19.6	22.8	NSSD
At least one orphan	32.2	20.1	44.3	
**Caring for a chronically ill household member**				
Not caring for a chronically ill household member	32.7	26.0	39.5	SSD
Currently caring for a chronically ill household member	20.6	18.9	22.3	
**Recent death**				
No recent death	19.9	18.3	21.5	NSSD
Any recent death	22.9	11.2	34.7	
**Effective dependency rate**				
Less than or equal to50%	27.9	24.8	31.1	NSSD
More than 50%	27.6	24.3	30.9	

*SSD – Statistically significant difference (p < 0.05); NSD – No statistically significant difference (p > 0.05). Descriptive statistics and 95% confidence interval are derived from descriptive analysis using the “Explore” tool in SPSS).*

### Coping strategies and household-level burden of care

Besides socio-demographic factors, other variables that may exacerbate a household’s food insecurity situation are the presence of AIDS orphans, effective dependency rate, recent occurrence of a death in the household, and caring for a chronically ill household member. We show in Table 3 the association between CSI and these explanatory variables. At the national level, households that care for orphans exhibited higher mean CSI score (32) compared with those with no orphans (21).

Similar to households with orphans, households with chronically ill individuals demonstrated less resilience to food insecurity. Households caring for chronically sick members have higher CSI (mean CSI of 32.7) in comparison to their counterparts without chronically ill members (mean CSI of 20.6) (Table 3).

## Discussion

The study assessed among others the negative coping skills that Ghanaian HIV-affected households adopt in ensuring their livelihoods. Our respondents were randomly selected from both rural and urban ART centers in the nation. We have presented the socio-demographic and health attributes household asset wealth, and coping strategies of 1,745 HIV-affected households in Ghana. Our analyses are descriptive in nature; and therefore encourage that caution be exercised when generalizations beyond this descriptive perspectives are done.

A greater proportion of them were educated. The higher attainment in formal education in HIV-positive households is likely to impact income earning opportunities and influence household food security and coping options [28]. Higher education in rural communities enhances peoples’ ability to participate in off-farm income activities, which is likely to increase household income and subsequently enhance access to food [29]. Polygynous unions are common in many parts of Africa [21]. The larger households (>5 members) reported in the study may actually be clusters of smaller nuclear families who then share the same lower risk factors as the households with less than five members.

This study is additionally important for its focus on food consumption coping strategies adopted by HIV-affected households. Households commonly use a combination of any of these 5 coping strategies to mitigate food insufficiency in their homes. More than half of these families reported limiting portion size, reducing number of meals eaten daily and relying on less expensive foods. Begging and harvesting immature crops were least employed coping strategies. As expected, asset rich households had the lowest CSI, and asset poor households reported the highest CSI. Asset poor households are more likely to engage in negative coping strategies than asset rich households because of less household income to support food expenditure and fewer physical assets of worth that can be sold in time of crisis (Figure 2).

Generally, the CSI scores were higher among female-headed households compared to their male counterparts. As in other countries in sub-Saharan Africa, women in Ghana mostly carry the responsibility of caring for the sick; they are thus unable to engage in economic activities outside of the home to earn a steady income. Ghanaian women tend to have lower educational attainment levels compared with men due to discriminatory access to formal education as children [30]. The lower attainment in formal education in households is likely to impact income earning opportunities [28] and push households to adopt negative strategies in an attempt to moderate food insufficiency problems. Higher education improves women’s ability to participate in higher income-generation activities which is likely to increase household income and subsequently enhance access to food [29].

The reported relationship between CSI and education level of the household head was mixed. Generally higher education of the household head was linked with lower CSI. However, in 4 regions, household heads with basic education had higher CSI than their uneducated peers. Not assessed in the current study, but nevertheless, significant in this discussion is the role of culture on coping mechanisms [8,9,31]. Ghana is politically partitioned into ten regions, and 216 districts. In each region, and in most districts are various cultural/socio-cultural practices. The influence of these culturally-determined practices on coping behaviors could be in dependent of one’s scholastic attainment. There is no argument that culture explains quite a lot of human behavioral tendencies and patterns. Sometimes referred to as individualism/collectivism, these cultural characteristics are related to different coping strategies [31]. See and Essau for example found that cultural values predicted coping, partly mediated by valuation of tradition, and cultural norms. Further research preferably employing both quantitative and qualitative techniques could provide a rewarding clarification to these relationships.

Based on the national average, HIV-affected households with AIDS orphans or chronically ill persons reported higher CSI, demonstrating the use of more negative food consumption coping strategies to buttress food security in these families (Table 3). Poor households with prime adult chronic illness are prone to food insecurity [32]. Households experiencing chronic illness and of prime-age adults suffer from loss of income and household labor shortages, which adversely affects food security due to declining agricultural productivity [33] and diminished household purchasing power [34]. Thus, asset rich households, regardless of a high-burden level of care may be resilient to food insecurity if the chronically ill persons are not the prime age adults, who are typically the main income earners in the household.

### Survey limitations

As is the usual in assessment of food security and vulnerability, the collection of market data is very critical, especially in settings characterized by instability/high food and fuel prices. In recent past, Ghana like many other countries in the sub-region fit this characterization. This phenomenon can impact negatively on household food security. The inability of the current survey to capture market data and subsequently provide necessary adjustments during the analysis is a limitation. Seasonality of food insecurity is a major problem in most parts of the country. Commonly referred to as the “lean season” and “harvesting season”, these periods respectively denote deterioration and amelioration of household state of vulnerability to food insecurity. Given that the data collection exercise was carried out during the course of the harvesting season, the level of food security could have been underestimated. In other words, households who were identified in this survey to be food secure during this period of the year could easily slip into food insecure during the lean season. As a consequence, the results of this assessment should be cautiously interpreted. These notwithstanding, it is unlikely that these limitations will significantly alter the main conclusions and recommendations.

## Conclusions

In summary, this paper suggests that HIV-affected households in Ghana do employ negative food consumption coping strategies to cushion food insufficiency and other pressures in their homes. While these strategies may provide short-term relief they are erosive, unsustainable, and undermine resilience in the long run. Reducing food intake, buying low quality cheap foods, gathering unusual kinds of wild foods, relying on casual labor, or going begging to battle food insufficiency, has dire implications for household members’ health, children’s school attendance and performance, and adults’ income-earning capacity in the long run.

There is an urgent need for policies that focus on building the capacity and stability of these households. Well-informed interventions that are appropriate for the local setting should aim to support HIV-affected households with long-term coping strategies that improve resilient to food insecurity.

## Endnote

^a^Defined as two or more epidemics interacting synergistically to contribute to excess burden of disease.

## References

[1] UNAIDS (2013). Global Report: UNAIDS report on the global AIDS epidemic 2013.

[2] UNAIDS (2011). UNAIDS World AIDS Day Report 2011.

[3] National AIDS/STI Control Programme (2012). National HIV prevalence and AIDS estimates report 2011-2015.

[4] National AIDS/STI Control Programme (2013). HIV Sentinel Survey Report 2012.

[5] Ghana AIDS Commission (2010). National HIV & AIDS Strategic Plan 2011-2015.

[6] Cooke G, Tanser F, Barnighausen T, Newell M-L (2010). Population uptake of antiretroviral treatment through primary care in rural South Africa. BMC Public Health.

[7] WHO, UNAIDS, UNICEF (2009). Towards Universal Access: scaling up priority HIV/AIDS interventions in the health sector. Progress Report 2009.

[8] Reddi A, Powers MA, Thyssen A (2012). HIV/AIDS and food insecurity: deadly syndemic or an opportunity for healthcare synergism in resource-limited settings of sub-Saharan Africa?. AIDS.

[9] Singer M, Clair S (2003). Syndemics and public health: reconceptualizing disease in bio-social context. Med Anthropol Q.

[10] Weiser SD, Tsai AC, Gupta R, Frongillo EA, Kawuma A, Senkungu J (2012). Food insecurity is associated with morbidity and patterns of healthcare utilization among HIV-infected individuals in a resource-poor setting. AIDS.

[11] Gillespie S, Kadiyala S (2005). HIV/AIDS and food and nutrition security: from evidence to action.

[12] de Waal A, Whiteside A (2003). New variant famine: AIDS and food crisis in southern Africa. Lancet.

[13] Greene JB (1988). Clinical approach to weight loss in the patient with HIV infection. Gastroenterol Clin North Am.

[14] Allard JP, Aghdassi E, Chau J, Salit I, Walmsley S (1998). Oxidative stress and plasma antioxidant micronutrients in humans with HIV infection. Am J Clin Nutr.

[15] Keating J, Bjarnason I, Somasundaram S, Macpherson A, Francis N, Price AB (1995). Intestinal absorptive capacity, intestinal permeability and jejunal histology in HIV and their relation to diarrhoea. Gut.

[16] Ivers LC, Cullen KA, Freedberg KA, Block S, Coates J, Webb P (2009). HIV/AIDS, undernutrition, and food insecurity. Clin Infect Dis.

[17] USAID (2011). HIV/AIDS health profile: West Africa.

[18] Oldewage-Theron WH, Dicks EG, Napier CE (2006). Poverty, household food insecurity and nutrition: coping strategies in an informal settlement in the Vaal Triangle, South Africa. Public Health.

[19] Ivers LC, Cullen KA (2011). Food insecurity: special considerations for women. Am J Clin Nutr.

[20] Gillespie S (2008). Food Prices and the AIDS Response. How they are linked, and what can be done.

[21] Gillespie S, Drimie S (2009). Seasonal dimensions of the HIV-hunger nexus in eastern and southern Africa. Seasonality Revisited International Conference; 8-10 July 2009; Institute of Development Studies, UK.

[22] Gillespie S, Kadiyala S, Greener R (2007). Is poverty or wealth driving HIV transmission. AIDS.

[23] Rawat R, Kadiyala S, McNamara P (2010). The impact of food assistance on weight gain and disease progression among HIV-infected individuals accessing AIDS care and treatment services in Uganda. BMC Public Health.

[24] De Waal A, Tumushabe J (2003). HIV/AIDS and food security in Africa.

[25] Ansell N, Robson E, Hajdu F, van Blerk L, Chipeta L (2009). The new variant famine hypothesis: moving beyond the household in exploring links between AIDS and food insecurity in southern Africa. Prog Dev Stud.

[26] WFP, CARE (2003). The Coping Strategies Index: Field Methods Manual.

[27] UNAIDS (2008). The Government of Zambia, WFP: Assessing Vulnerability of HIV-affected Households in Three Urban Areas in Zambia.

[28] Maxwell D, Levin C, Armar-Klemesu M, Ruel M, Morris S, Ahiadeke C (2000). Urban livelihoods and food and nutrition security in Greater Accra, Ghana.

[29] Dose, Henriette. Securing Household Income among Small-Scale Farmers in Kakamega District: Possibilities and Limitations of Diversification. GIGA Working Paper No. 41. GIGA German Institute of Global Area Studies. Hamburg, Germany; 2007.

[30] Safo-Kantanka AB, Attah E, Ofosu E, Nagel J, Akuto MA, van Beurden M (2006). Shifting trends in rural livelihood: a case study of Asutifi District.

[31] See CM, Essau CE. Coping strategies in cross-cultural comparison. In: Mayer B, Kornadt HJ (Eds). Psychologie – Kultur – Gesellschaft. VS Verlag für Sozialwissenschaften. 2010:161-173.

[32] Kaschula S (2011). Using people to cope with the hunger: social networks and food transfers amongst HIV/AIDS afflicted households in KwaZulu-Natal, South Africa. AIDS Behav.

[33] Jayne TS, Villarreal M, Pingali P, Hemrich G, Gillespie S (2006). HIV/AIDS and the agricultural sector in Eastern and Southern Africa: anticipating the consequences. AIDS, poverty, and hunger: challenges and responses Highlights of the International Conference on HIV/AIDS and Food and Nutrition Security, Durban, South Africa, 14-16 April, 2005.

[34] Masuku MB, Sithole MM (2009). The impact of HIV/AIDS on food security and household vulnerability in Swaziland. Agrekon.

